# Implementation of healthy eating and physical activity practices in Australian early childhood education and care services: A cross-sectional study

**DOI:** 10.1016/j.pmedr.2023.102455

**Published:** 2023-10-04

**Authors:** Melanie Lum, Alice Grady, Luke Wolfenden, Christophe Lecathelinais, Sze Lin Yoong

**Affiliations:** a Global Centre for Preventive Health and Nutrition, Institute for Health Transformation, Deakin University, School of Health and Social Development, Faculty of Health, Geelong VIC 3220 Australia; b School of Medicine and Public Health, University of Newcastle, Callaghan, NSW 2308 Australia; c Population Health, Hunter New England Local Health District, Locked Bag 10, Wallsend NSW 2287 Australia; d National Centre of Implementation Science, University of Newcastle, Locked Bag 10, Wallsend NSW 2287 Australia

**Keywords:** Early childhood education and care, Healthy eating, Physical activity, Policy, Practice, Health promotion, Public health

## Abstract

**Background:**

The monitoring of healthy eating and physical activity environments is recommended to support population health. Early childhood education and care (ECEC) services are a key setting to deliver obesity prevention strategies as they reach the majority of children aged under five years and have existing supporting infrastructure.

**Objectives:**

The aim of this study was to i) describe the prevalence of implementation of evidence-based healthy eating and physical activity practices in an Australian sample of ECEC services, and ii) examine associations between implementation of practices and service characteristics.

**Methods:**

A random sample of 2,100 centre-based ECEC services across Australia were invited to participate in a cross-sectional survey online or via telephone (August 2021-April 2022). Service characteristics and implementation of 18 evidence-based healthy eating and physical activity practices were assessed. Descriptive statistics and linear mixed regression analyses were performed.

**Results:**

A total of 1,028 (51.8%) eligible services participated. The healthy eating and physical activity practices with the lowest prevalence of implementation were related to encouraging children to consume age-appropriate beverages (17.9%) and providing families with child physical activity education via workshops or meetings (14.6%), respectively. There was a statistically significant difference in the implementation of healthy eating practices by service characteristics, with long day care services implementing significantly more practices than preschools (p < 0.01).

**Conclusion:**

There is room to improve the implementation of evidence-based healthy eating and physical activity practices ECEC services. Implementation efforts should be directed towards preschools.

## Highlights

• Evidence-practice gaps exist for some obesity prevention practices in ECEC services.
• Investment is needed to support obesity prevention practices in ECEC services.
• Preschools may need additional support to implement healthy eating practices.
• Implementation of practices did not differ by service socioeconomic status.

## 1 Introduction

A nutritious diet and sufficient physical activity in childhood can protect against the development of obesity and related diseases over the lifetime. (Hidayat et al., 2020, Ambrosini, 2014) As such, governments and authorities in countries such as the United States, (U.S. Department of Agriculture, 2020, U.S. Department of Health and Human Services, 2018) Australia (Department of Health and Aged Care, 2021, National Health and Medical Research Council, 2013) and the United Kingdom (Public Health England, 2016, UK Chief Medical Officer (CMO) Guidelines Writing Group, 2019) have developed recommendations for children aged 2–6 years to achieve optimal dietary and activity outcomes. Despite this, evidence internationally suggests the majority of children do not meet these recommendations. (Fox et al., 2016, Banfield et al., 2016, Public Health England, 2018, Australian Institute of Health and Welfare, 2018, Tucker, 2008, Tapia-Serrano et al., 2022) For example, 70–99% of children aged 2–3 years in both the United States and Australia do not meet nationally recommended intakes for vegetables, nor wholegrains. (Fox et al., 2016, Australian Institute of Health and Welfare, 2018) Further, systematic review evidence from 13 countries indicates 89% of children aged 3–5 years do not meet physical activity, sedentary behaviour and sleep guidelines endorsed by the World Health Organization. (Tapia-Serrano et al., 2022).

Settings-based approaches provide the opportunity to support the development of child healthy eating and physical activity at a population-level, and are recommended by the World Health Organization. (World Health Organisation, 2012) Early childhood education and care (ECEC) represents an attractive setting to improve healthy eating and physical activity in young children as 87% of 3–5 year olds attend these services globally (OECD, 2021) and they have the organisational structure to support children’s health behaviours. Several jurisdictions in high-income countries have developed best-practice guidelines around healthy eating and physical activity for ECEC (Jackson et al., 2021) and many countries have accreditation standards requiring services to support and promote child health behaviours. (Australian Children's Education and Care Quality Authority, 2018, American Academy of Pediatrics APHA, 2019).

The implementation of healthy eating and physical activity practices in the ECEC setting to improve child health behaviours is also supported by the evidence base. (Matwiejczyk et al., 2018, Stacey et al., 2017, Lum et al., 2022, Yoong et al., in press) A recent Cochrane review found interventions delivered in ECEC services improve child diet quality, fruit consumption and vegetable consumption. (Yoong et al., in press) A recent umbrella review of physical activity interventions in the ECEC setting found interventions involving the provision of opportunities for physical activity, educator training, a physical activity-promoting environment or family involvement, resulted in improved child physical activity (Lum et al., 2022).

To date, few studies have reported on the implementation healthy eating and physical activity practices in nationally representative samples of ECEC services. In the US, a survey conducted in 2008 with 1,583 ECEC indicated services implemented an average of 23 of 30 assessed healthy eating and physical activity practices. (Whitaker et al., 2009) In England, 2,000 randomly selected nurseries were assessed against nutrition guidelines in 2012–2013, with over 50% of services meeting 13 of 20 nutrition guidelines around foods served and healthy eating practices. (Neelon et al., 2015) Both studies noted that measurement of implementation was challenging due to poorly defined recommendations for the setting, which are not expressed in a clear quantitative, nor measurable, manner. (Whitaker et al., 2009, Neelon et al., 2015).

To the author’s knowledge, just one study has been published describing the implementation of evidence-based nutrition or physical activity practices in a nationwide sample of Australian ECEC services. Jones and colleagues (Jones et al., 2020) conducted an online or telephone survey with 326 ECEC service managers in 2017–2018 to assess the implementation of a single physical activity practice (continuous outdoor free play schedules) in centre-based ECEC services across Australia. It was reported 62% of services implemented continuous outdoor free play sessions for an average of three periods per day for 126 min per period. These findings highlight a need to be understand implementation of different practices beyond the one examined in this study. To inform national priorities and provide a benchmark of progress in the sector, the monitoring of obesity prevention practice implementation in a nationally representative sample of ECEC services is necessary. Previous research conducted in Australia and the United States indicates service characteristics, such as socioeconomic status, locality, quality rating and size may be associated with implementation of practices. (Jones et al., 2020, Green et al., 2020, Sisson et al., 2012) An examination of differences in implementation by service characteristics may provide insight into types of services which may require additional implementation support.

The primary aim of this study is to describe the prevalence of implementation of evidence-based healthy eating and physical activity practices in ECEC centres nationally. Secondary aims are to examine the associations between implementation of healthy eating and physical activity practices and service characteristics, including locality (“rural” vs “urban”), socioeconomic status (“low” vs “high”), service type (preschool vs long day care), rating for Quality Area 2: Childrens’ health and safety (“working towards NQS” vs “meeting NQS” vs “exceeding NQS”) and service size (“small” vs “large”).

## 2 Materials and methods

This study received ethical approval from the Hunter New England (reference 06/07/26/4.04) and the University of Newcastle (reference H-2008–0343) human research ethics committees.

### 2.1 Study design, setting and eligibility

A cross-sectional survey, completed via telephone or online, was conducted with Nominated Supervisors (or an elected staff member) of centre-based ECEC services across Australia (August 2021-April 2022). Currently operating preschool or long day care service were eligible. Services were ineligible if they were part of a Department of Education primary or central school, a family day care service, temporarily closed, exclusively provided outside-of-school-hours care or catered solely to children with additional needs. Reasons for exclusion were due to different operational characteristics from our target sample or the requirement for different ethical approval. Participants were required to have sufficient English to complete the survey.

There are approximately 16,500 centre-based services registered across Australia, which includes preschools and long day care services which care for children aged between 0 and 6 years. (Australian Children's Education and Care Quality Authority, 2022) Services may care for specific age groups (e.g. 3–6 years), and may provide main meals to children (i.e. menu services) or have families pack meals for children (i.e. lunchbox services).

### 2.2 Sample and recruitment

A list of all operational centre-based ECEC services was obtained from the publicly available Australian Children’s Education & Care Quality Authority (ACECQA) register. (Australian Children’s Education and Care Quality, 2021) Services pre-identified as ineligible were removed. The remaining 10,578 services served as the sampling frame for the study. We used stratified sampling approaches using the SURVEYSELECT procedure in SAS to select a representative sample of 2,100 services (approximately 20% of total services were randomly selected), with state as the stratum. This was consistent with recommendations to ensure representative sampling for population-based surveys. (Martínez-Mesa et al., 2016) Services were mailed a hardcopy and an email version of the study information statement and invited to participate in a 30 min online survey involving a series of questions, including around service characteristics and current implementation of evidence-based healthy eating and physical activity policies and practices in ECEC. A reminder email was sent one week following the initial invitation to services yet to respond. Trained telephone interviewers also contacted services which had not yet completed the survey online and invited services to complete the survey via telephone. Non-responding services were contacted between two and 10 times.

Nominated Supervisors were the preferred survey responders as they are responsible for the management and leadership of an ECEC service, and for ensuring each child’s healthy eating and physical activity is promoted. (Australian Children's Education and Care Quality Authority, 2018) The Nominated Supervisor was able to elect another staff member with a strong knowledge of the services operations to complete the survey, either online or via telephone, on behalf of the service if this was more appropriate. As specified in the information statement, participation in the survey was considered indication of consent.

To reduce participant burden, services were randomised to receive survey items relating either to healthy eating or physical activity using a randomisation function in SAS.

### 2.3 Sample size

Assuming approximately 50% consent and accounting for 5% ineligibility, the initial sample size of 2,100 would allow for approximately 500 participants to complete healthy eating-related, and 500 participants to complete physical activity-related, survey items. This provides a weighted national prevalence of practice implementation with 95% precision of ± 4.38% for both healthy eating and physical activity practices.

### 2.4 Measures

#### 2.4.1 Service characteristics

All ECEC services operating within Australia are required to be registered with ACECQA. (Australian Children's Education and Care Quality Authority, 2018) ACECQA assesses and rates seven NQS for each service: “working towards NQS”, the lowest rating which indicates the service provides a safe education program; “meeting NQS” indicates the service meets the NQS; and “exceeding NQS”, the highest rating which indicates the service goes beyond the requirements of the NQS. (Australian Children's Education and Care Quality Authority, 2018) Quality Area 2 refers to children’s health and safety standards, and in part, requires services to support and promote healthy eating and physical activity for children. The assessment of this varies from state to state, however, it broadly requires services to create healthier nutrition and activity environments for children attending care to align with state-specific guidelines around provision of healthier food, opportunities for play, education-based approaches and working in partnership with families. Quality Area 2 rating information was obtained from the ACECQA register, as were service postcodes to determine the socioeconomic status and locality of participating ECEC services. (Australian Children’s Education and Care Quality, 2021) Services were classified as “low” (i.e. lower 50%) or “high” (i.e. higher 50%) socioeconomic status as ranked by the Australian Socioeconomic Indices for Areas 2016 (Australian Bureau of Statistics., 2033) and as “urban” (i.e. major cities) or “rural” (i.e. inner/outer regional, remote or very remote) according to the Australian Statistical Geography Standard 2021. (ASGS, 2021) To obtain additional service characteristic information, the survey included items assessing: service operation type (i.e. preschool or long day care); hours of operation; age groups cared for; number of children attending daily; total number of non-English speaking children enrolled; and number of full-time, part-time and casual educators employed. Service size was determined based on the median number (median = 55) of children attending daily, as reported by participants in this study. Services were categorised as “small” if <55 children attended daily, and “large” if 55 or more children attended daily. One item assessed the responders’ current role.

#### 2.4.2 Evidence-based healthy eating and physical activity practices

We undertook a systematic process to identify practices assessed as both evidence-based and recommended by guidelines to improve child dietary intake and physical activity in care based on evidence from systematic and umbrella reviews of studies conducted in the ECEC setting. (Jackson et al., 2021, Lum et al., 2022, Yoong et al., in press) We identified 10 healthy eating and eight physical activity evidence-based and recommended practices to provide the best opportunity for child health, most of which were multicomponent and involved time or frequency parameters. Services were assessed as either “fully”; “partially” or “not” implementing each practice, according to criteria (Table 1). Criteria for “full” implementation encompassed all strategies to the recommended dose and frequency, which are likely to have the greatest impact on improving child healthy eating or physical activity, based on the evidence. Criteria for “partially” meeting included the practice components at a reduced level as defined by the research team. This level may not be at the recommended dose or frequency outlined in the literature, however, can indicate whether services need support to initiate the implementation of a practice, or instead require support to increase the frequency or quality of their current practices. Services were classed as “not” implementing if they did not meet criteria for “full”, nor “partial”, implementation.

**Table 1:** Recommended and evidence-based healthy eating and physical activity practices and criteria to meet “full” or “partial” implementation.

**Recommended evidence-based practice**	**Criteria to meet “full” implementation*(all criteria must be met)***	**Criteria to meet “partial” implementation*(all criteria must be met)***
**Healthy eating**		
**Service has a written nutrition policy**	• Service has written policy, procedure or guideline encouraging child healthy eating which includes the following elements: o Strategies are in place to ensure that foods provided by families is consistent with the Australian Dietary Guidelines *(lunchbox services only)* o Food provided by the service is consistent with the Australian Dietary Guidelines *(menu services only)* o Food isn't used as a reward or incentive for children o Educators role model healthy food and drink choices o Creating healthy mealtime environments o Educator practices to encourage healthy eating o Planned and informal nutrition education for children o Professional development on child nutrition o Guidelines for foods offered during holidays and celebrations	• Service has written policy, procedure or guideline encouraging child healthy eating which includes the following elements: o Strategies are in place to ensure that foods provided by families is consistent with the Australian Dietary Guidelines *(lunchbox services only)* o Food provided by the service is consistent with the Australian Dietary Guidelines *(menu services only)*
**Service supports menu planning according to dietary guidelines***Applicable to menu services which have a service cook only*	• Service cook completed training in planning, preparing and serving nutritious meals and snacks in the past 2 years • Service cook uses nutrition guidelines to plan menus • Service cook is provided with menu planning support (minimum of 1): o Support from external experts such as health promotion team or dietitian o Written resources such as recipes or books	• Service cook uses nutrition guidelines to plan menus • Service cook is provided with menu planning support (minimum of 1): o Support from external experts such as health promotion team or dietitian o Written resources such as recipes or books
**Service provides sufficient serves of fruit and vegetables per child, per day for children aged 6-24 months***Applicable to menu services only*	• Children aged 6-24 months are provided with at least 1 serve of fruit per day • Children aged 6-24 months are provided with at least 2 serves of vegetables per day	• Children aged 6-24 months are provided with at least 1 serve of fruit per day • Children aged 6-24 months are provided with at least 1 serve of vegetables per day
**Service provides sufficient serves of fruit and vegetables per child, per day for children aged 2-6 years and limits discretionary food items***Applicable to menu services only*	• Children aged 2-6 years are provided with at least 1 serve of fruit per day • Children aged 2-6 years are provided with at least 2 serves of vegetables per day, over 2 meals • Service has not provided discretionary foods (e.g. chocolate, processed meats, cakes, chips, flavoured or sweet biscuits/crackers, rolls ups, jelly or muesli bars) in the past two weeks	• Children aged 2-6 years are provided with at least 1 serve of fruit per day • Children aged 2-6 years are provided with at least 1 serve of vegetables per day, over 1 meal • Service has not provided more than 1 type of discretionary food in the past two weeks
**Service encourages children to consume age-appropriate beverages**	• Service provides water to children, and may provide reduced fat milk to children aged 2-6 years • Service does not provide sugar-sweetened beverages to children (e.g. fruit juice, cordial, flavoured milk or soft drink) • Service implements strategies to encourage children to consume age-appropriate beverages including water and milk every day • Educators role model healthy drink choices • Service has an appropriate beverage policy, which includes the following elements: o Supporting water as a drink for children aged 3-5 years o Drinks provided/allowed by the service are consistent with the Australian Dietary Guidelines or Caring for Children Guidelines o Sugar-sweetened beverages are not available to children	• Service provides water to children, and may provide reduced fat milk or full cream milk aged 2-6 years • Service does not provide sugar-sweetened beverages to children • Service implements strategies to encourage children to consume age-appropriate beverages including water and milk at least weekly • Educators role model healthy drink choices • Service has an appropriate beverage policy which includes the following elements: o Supporting water as a drink for children aged 3-5 years o Drinks provided/allowed by the service are consistent with the Australian Dietary Guidelines or Caring for Children Guidelines o Sugar-sweetened beverages are not available to children
**Service supports families to pack lunchboxes according to guidelines***Applicable to lunchbox services only*	• Service provides a resource to families which refers to the Australian Dietary Guidelines and include recommendations for the types of food and drinks brought in lunchboxes from home • Service observes children's lunchboxes to ensure that they are consistent with the Australian Dietary Guidelines every day • Service provides feedback to families when lunchboxes are not consistent at least weekly	• Service provides a resource to families which refers to the Australian Dietary Guidelines and include recommendations for the types of food and drinks brought in lunchboxes from home • Service observes children's lunchboxes to ensure that they are consistent with the Australian Dietary Guidelines at least weekly • Service provides feedback to families when lunchboxes are not consistent at least monthly
**Service provides various healthy eating education to children**	• Service provides each of the following healthy eating activities to children at least weekly: o Planned healthy eating education o Interactive, experiential healthy eating activities o Intentional exposure to different vegetables as part of experiential learning o Play based healthy eating activities	• Service provides each of the following healthy eating activities to children at least monthly: o Planned healthy eating education o Interactive, experiential healthy eating activities o Intentional exposure to different vegetables as part of experiential learning o Play based healthy eating activities
**Staff role-model healthy eating during mealtimes**	• Staff model, reinforce and implement healthy eating and nutrition practices with children during mealtimes every day (e.g. sitting with the children during mealtimes, eating healthy foods in front of the children, talking with the children about the foods they are eating)	• Staff model, reinforce and implement healthy eating and nutrition practices with children during mealtimes weekly
**Educators are trained and supported to promote healthy eating for children**	• All primary contact educators have participated in professional development or training which promotes healthy eating for children in the past year • Service provides educators with additional support or resources to increase healthy eating for children (minimum of 3): o Refresher training o Newsletters o Feedback from service or other organisations o Telephone support	• At least half of the primary contact educators have participated in professional development or training which promotes healthy eating for children in the past year • Service provides educators with additional support or resources to increase healthy eating for children (minimum of 3): o Refresher training o Newsletters o Feedback from service or other organisations o Telephone support
**Service provides families with child healthy eating education via workshops or meetings**	• Services offers healthy eating education to all families of children of all ages in the form of parent/families workshops or meetings annually	• Services offers healthy eating education to families of children aged 3-6 years in the form of parent/families workshops or meetings annually
**Physical activity**		
**Service has a written physical activity policy**	• Service has written policy, procedure or guideline encouraging physical activity which includes the following elements: o Reference to the Australian 24-Hour Movement Guidelines for the Early Years (Birth to 5 years) o Physical activity is embedded in the daily curriculum through spontaneous and intentionally planned active, play time that is both child initiated and educator led o Educators actively role model appropriate physical activity behaviours to children o Professional development on children’s physical activity o Education for families on children's physical activity	• Service has written policy, procedure or guideline encouraging physical activity which includes the following elements: o Reference to the Australian 24-Hour Movement Guidelines for the Early Years (Birth to 5 years)
**Services provides opportunities for child physical activity every day**	• Children aged 3-6 years are provided the opportunity for physical activity (including child-initiated or educator-led) for a minimum of 50% of opening hour • Physical activity opportunities include at least 120 minutes of unstructured free play and 60 minutes of educator-led activities	• Children aged 3-6 years are provided the opportunity for physical activity (including child-initiated or educator-led) for a minimum of 50% of opening hours
**Service promotes physical activity during outdoor play time every day**	• Service provides children a minimum of 120 minutes of outdoor play time every day • Service plans specific physical activities for outdoor time every day	• Service provides children a minimum of 120 minutes of outdoor play time every day
**Service creates a physical environment which promotes child physical activity**	• Service has sufficient portable play equipment for children to share, which is available and in good condition for children to use every day • Service reorganises the outdoor play area at least weekly	• Service has sufficient portable play equipment for children to share, which is available and in good condition for children to use every day • Service reorganises the outdoor play area at least monthly
**Educators provide an activity designed to intentionally teach and develop the various fundamental movement skills for children aged 3-6 years of age**	• Service provides an activity designed to intentionally teach and develop fundamental movement skills for children aged 3-6 years of age every day	• Service provides an activity designed to intentionally teach and develop fundamental movement skills for children aged 3-6 years of age at least 3 days per week
**Educators model and engage children in active play**	• Educators model and engage children in active play every day	• Educators model and engage children in active play at least multiple times per week
**Educators are trained and supported to promote physical activity for children**	• All primary contact educators have participated in training which promotes physical activity for children in the past year • Service provides educators with additional support or resources to increase physical activity for children (minimum of 3): o Peer-support meetings with colleagues o Refresher training o Newsletters o Feedback from service or other organisations o Telephone support	• At least half of the primary contact educators have participated in training which promotes physical activity for children in the past year • Service provides educators with additional support or resources to increase physical activity for children (minimum of 3): o Peer-support meetings with colleagues o Refresher training o Newsletters o Feedback from service or other organisations o Telephone support
**Service provides families with child physical activity education via workshops or meetings**	• Service offers physical activity education to all families in the form of parent/families workshops or meetings annually	• Service offers physical activity education to families of children aged 3-6 years in the form of parent/families workshops or meetings annually

Items to assess the implementation of these practices were developed by the research team based on valid and reliable audit tools to assess the healthy eating and physical activity environment in ECEC settings identified in a systematic review (Appendix A). (Ajja et al., 2015) These tools are commonly used and adapted to assess implementation of healthy eating and physical activity practices in centre-based ECEC via self-report survey when the use of objective measures (e.g. direct observation) is not feasible. (Yoong et al., 2016) We obtained feedback around item comprehension from stakeholders, including health promotion staff, researchers, dietitians and state health organisations and piloted the survey with members of the research team and trained telephone interviewers prior to finalising items. Multiple items were used to assess each practice, as per previous tools used to measure implementation via survey. (Ajja et al., 2015).

### 2.5 Analyses

Statistical analyses were performed using SAS 9.3 statistical software. Descriptive statistics were used to describe service characteristics and implementation of practices. Where data was missing for items required to make assessments on implementation, these services were removed from the analysis for the respective practice. Comparisons of characteristics (locality and socioeconomic classification) of non-consenters and consenters were undertaken using logistic regression models.

We performed multivariable linear regression analyses, controlling for state, to examine differences in implementation of evidence-based practices by service characteristics including locality, service type, quality area, and service size. Previous research indicates these characteristics to be associated with implementation of practices (Jones et al., 2020, Green et al., 2020, Sisson et al., 2012) or are expected to be correlated with implementation. (Australian Children's Education and Care Quality Authority, 2018) For services that provided sufficient data to determine implementation (i.e. at least 50% of healthy eating (n = 5) or physical activity (n = 4) practices), the percentage of healthy eating or physical activity practices “fully” implemented was determined. This was calculated as the number of practices considered to be “fully” implemented by the service divided by the total number of assessable practices, times by 100. Statistical significance was set at p < 0.05 for all tests.

## 3 Results

### 3.1 Sample

A sample of 2,100 services were contacted. Following contact, 116 services were ineligible due to: not currently operating (n = 14); being a Department of Education primary or central school (n = 52); solely catering to children with additional needs (n = 21); or not providing sufficient information to judge eligibility (n = 29). Of the remaining 1,984 eligible services, 956 did not consent to participate: 685 refused; 12 were non-contactable; and 259 did not attempt the survey following phone contact. A total of 1,028 (51.8%) eligible services consented to participate: 576 (56.0%) participated in the survey via telephone and 452 (44.0%) participated via online. There were 535 (52.0%) services randomised to receive items around healthy eating and 493 (48.0%) around physical activity. There was a statistically significant lower proportion of consenting services located in urban areas, compared to rural areas (49.7% vs 58.6%, p < 0.01) and high socioeconomic areas compared to low (49.2% vs 55.8%, p < 0.01) than non-consenters.

### 3.2 Characteristics

Responders were most frequently Nominated Supervisors (55.1%).The majority of responding services were long day care services (90.0%), located in high socioeconomic areas (56.5%), and in urban areas (72.8%). Services were open for an average of 10.9 h per day and cared for on average 58 children daily (Table 2).

**Table 2:** Characteristics of responding early childhood education and care services across Australia in 2021–2022.

**Characteristic**	**Responding services, n (%)** **(N = 1,028)**
Main position of responder^a^	
Nominated supervisor	563 (55.1)
Director	311 (30.4)
Educator	22 (2.2%)
Room leader	21 (2.1%)
Service owner	9 (0.9%)
Other	96 (9.4%)
Service type	
Preschool	103 (10.0)
Long day care	925 (90.0)
State	
Australian Capital Territory	19 (1.8)
New South Wales	438 (42.6)
Northern Territory	14 (1.4)
Queensland	206 (20.0)
South Australia	41 (4.0)
Tasmania	15 (1.5)
Victoria	193 (18.8)
Western Australia	102 (9.9)
Locality	
Rural	280 (27.2)
Urban	748 (72.8)
Socioeconomic status of service area^b^	
High	580 (56.5)
Low	447 (43.5)
Age groups cared for	
0–2 years	904 (87.9)
3–6 years	994 (96.7)
Above 6 years	158 (15.4)
Service operates 5 days per week	1008 (98.1)
Opening hours per day (mean, SD)^b^	10.9 (1.3)
Number of children attending daily (mean, SD)^c^	57.8 (29.7)
Service has children of non-English speaking background^d^	794 (78.5)
Number of full-time educators (mean, SD)^d^	9.2 (8.0)

a N = 1022

b N = 1027

c N = 1026

d N = 1012

### 3.3 Prevalence of implementation of healthy eating and physical activity practices

The prevalence of “full”, “partial” and “not” implementing practices is described in Table 3. For healthy eating practices, “full” implementation was most common for ‘staff role-modelling around healthy eating during mealtimes’ (95.4%) and least common for ‘encouraging children to consume age-appropriate beverages’ (14.8%).

**Table 3:** Prevalence of responding Australian ECEC service’s implementation of healthy eating and physical activity practices in 2021–2022.

**Recommended evidence-based healthy eating practice**	**N**	**Services “fully” implementing** **n (%)**	**Services “partially” implementing** **n (%)**	**Services “not” implementing** **n (%)**
Service has a written nutrition policy	523	193 (36.9)	264 (50.5)	66 (12.6)
Service supports menu planning according to dietary guidelines *(menu services which have a service cook only*	363	259 (71.4)	73 (20.1)	301 (8.5)
Service provides sufficient serves of fruit and vegetables per child, per day for children aged 6–24 months *(menu services only)*	110	67 (60.9)	33 (30.0)	10 (9.1)
Service provides sufficient serves of fruit and vegetables per child, per day for children aged 2–6 years and limits discretionary food items *(menu services only)*	415	136 (32.8)	171 (41.2)	108 (26.0)
Service encourages children to consume age-appropriate beverages	526	94 (17.9)	237 (45.0)	195 (37.1)
Service supports families to pack lunchboxes according to guidelines (*Lunchbox services only)*	115	40 (34.8)	31 (27.0)	44 (38.3)
Service provides various healthy eating education to children	500	139 (27.8)	149 (29.8)	212 (42.4)
Staff role-model healthy eating during mealtimes	525	501 (95.4)	19 (3.6)	5 (1.0)
Educators are trained and supported to promote healthy eating for children	508	131 (25.8)	114 (22.4)	263 (51.8)
Service provides families with child healthy eating education via workshops or meetings	525	146 (27.8)	8 (1.5)	371 (70.7)
**Recommended evidence-based physical activity practice**	**N**	**Services “fully” meeting** **n (%)**	**Services “partially” meeting** **n (%)**	**Services “not” meeting** **n (%)**
Service has a written physical activity policy	479	141 (29.4)	79 (16.5)	259 (54.1)
Services provides opportunities for child physical activity every day	464	336 (72.4)	62 (13.4)	66 (14.2)
Service promotes physical activity during outdoor play time every day	482	115 (23.9)	75 (15.6)	292 (60.6)
Service creates a physical environment which promotes child physical activity	480	291 (60.6)	81 (16.9)	108 (22.5)
Educators provide an activity designed to intentionally teach and develop the various fundamental movement skills for children aged 3–6 years of age	458	370 (80.8)	37 (8.1)	51 (11.1)
Educators model and engage children in active play	480	369 (76.9)	63 (13.1)	48 (10.0)
Educators are trained and supported to promote physical activity for children	464	79 (17.0)	99 (21.3)	286 (61.6)
Service provides families with child physical activity education via workshops or meetings	480	70 (14.6)	9 (1.9)	401 (83.5)

For physical activity practices “full” implementation was most frequent for ‘providing an activity designed to intentionally teach and develop the various fundamental movement skills for children aged 3–6 years of age’ (80.8%) and lowest for ‘providing families with child physical activity education via workshops’ (14.6%).

Based on 525 services with sufficient data, services “fully” implemented a mean of 41.3% (SD 19.3) healthy eating practices. Of the 480 services with sufficient data on physical activity practices, services “fully” implemented a mean of 46.8% (SD 19.7) physical activity practices.

### 3.4 Associations between healthy eating and physical activity practices and service characteristics

Long day care services implemented a significantly higher mean percentage of healthy eating practices in comparison to preschools (42.3% vs 33.3%, p < 0.001) (Table 4). No statistically significant associations were found between healthy eating practice implementation and locality, socioeconomic status of service area, service size or Quality Area 2 rating. No statistically significant associations were found between physical activity practice implementation and any service characteristics assessed.

**Table 4:** Association between Australian ECEC service characteristics and percentage of practices “fully” implemented in 2021–2022.

**Service characteristics**	**Healthy eating practices (n = 525),** **mean (SD)**	**Effect size** **(MD)**	**p-value**	**Physical activity practices (n = 480),** **mean (SD)**	**Effect size** **(MD)**	**p-value**
Type			**<0.01**			0.71
Long day care	42.29 (19.15)	11.27		46.81 (19.81)	1.27	
Preschool	33.26 (18.88)			46.35 (19.19)		
Locality			0.07			0.55
Rural	39.27 (20.14)	−4.20		47.59 (18.32)	−1.42	
Urban	42.08 (18.95)			46.47 (20.25)		
SES of service area			0.76			0.62
High	41.40 (19.77)	−0.60		47.27 (19.99)	0.99	
Low	41.18 (18.73)			46.23 (19.42)		
Size			0.21			0.93
Large	42.00 (19.32)	2.30		47.08 (19.44)	0.18	
Small	40.65 (19.32)			46.44 (20.08)		
Quality Area 2 Rating			0.08			0.44
Exceeding NQS	44.64 (19.14)	7.59		47.25 (17.69)	1.15	
Meeting NQS	40.64 (19.24)			46.59 (20.03)		
Working toward NQS	37.33 (21.37)			44.43 (18.83)		

SD: standard deviation; MD: mean difference; SES: socioeconomic status; NQS: National Quality Standard.

## 4 Discussion

This study provides important insights into the current “full” and “partial” implementation of recommended evidence-based healthy eating and physical activity policies and practices in centre-based ECEC services across Australia. Broadly, we found a large variation in the prevalence of implementation across practices. Association analyses indicate long day care services implement a significantly higher percentage of healthy eating practices than preschools.

There was a low prevalence of services (18%) reporting “full” implementation ‘encouraging children to consume age-appropriate beverages’. This contrasts previous research conducted in the United States and Australia, which indicate practices around beverages are implemented by over 90% of services. (Sisson et al., 2012, Wolfenden et al., 2015) This may be partly explained by our multicomponent criteria, which required services to have an beverage policy, role-model healthy drink choices, and serve reduced fat milk to children as per national dietary guidelines, (National Health and Medical Research Council, 2013) while previous research measures have only considered provision of sugar-sweetened beverages and water. This suggests services may require support to meet current evidence-based and recommended levels of implementation of this practice. The majority of services did “not” promote physical activity during outdoor play every day (61%). Several studies have found educators commonly report safety, supervision requirements and inclement weather conditions as barriers to provision of outdoor play in ECEC. (Coleman and Dyment, 2013, Ernst, 2014, Little and Wyver, 2008) Efforts to address these barriers, including staffing support or resources to ease concerns regarding child safety, may be required to improve implementation of this practice.

For both healthy eating and physical activity, prevalence of “full” implementation of educator training and providing families with educational workshops was low (15–28%) with most services (52–84%) “not” meeting these practices. Comparatively, previous cross-sectional studies assessing implementation via self-report survey conducted in the United States and Australia, suggest prevalence of implementation of these practices may be higher, ranging from 46 to 77%. (Sisson et al., 2012, Yoong et al., 2016, Liu et al., 2016, Byrd-Williams et al., 2019) It should be noted that data collection for this study took place in 2021–2022 and multiple sporadic lockdown orders occurred during this time, affecting states at varying periods and lengths of duration. (Department of Health and Aged Care, 2021) This likely impacted the ability to conduct face-to-face training, workshops or meetings. Nevertheless, the implementation of these practices is important to support child health behaviours. (Stacey et al., 2017, Lum et al., 2022) Follow-up research should be undertaken in a national sample to assess whether such activities have since returned.

This study found long day care services implemented a significantly higher proportion of healthy eating practices compared to preschools. There are several key characteristics which vary between preschool and long day care services, including shorter opening hours, catering to older children, specific focus on school readiness and smaller service sizes. (Australian Children's Education and Care Quality Authority, 2018) Within our sample (data not shown), preschool services were more likely to be lunchbox services and open for three hours shorter than long day care services, aligning with state and national data. (Barnes et al., 2021, Australian Bureau of Statistics, 2022) Given menu services were assessed against two additional practices compared to lunchbox services, this may partly explain findings. Nonetheless, given child dietary intake has been shown to be significantly associated with healthy eating practices in lunchbox services, (Barnes et al., 2021) investment in implementation support for these service types is warranted. Further examination of factors which may influence preschools’ implementation of healthy eating practices, particularly in relation to lunchboxes, may improve understanding of this finding and guide future implementation support efforts.

This study did not identify any statistically significant associations between implementation of practices and service locality, socioeconomic area, size or Quality Area 2 rating. Previous research on the associations of service characteristics and implementation of practices in Australia has been mixed. (Jones et al., 2020, Green et al., 2020, Sisson et al., 2012, Yoong et al., 2016) Many state-wide population-based programs supporting ECEC (e.g. Munch & Move, Achievement Program) have strategies in place to support more equitable reach of health promotion support. It is possible this may have resulted in similar reach across services located in different socioeconomic areas.

A strength of this study was the inclusion of a national sample and assessment of implementation of full and partial recommended practices which are supported by the evidence-base. Nevertheless, the consent rate was just above 50%, and therefore it is possible participation bias may have occurred. There was a significant difference between participants and non-participants in locality and socioeconomic classification, potentially limiting the generalisability of these findings. While items were based on valid and reliable tools and reviewed by stakeholders, the measure was not validated and relied on self-report. Data was also collected using two modalities to increase the accessibility to participants, however, post-hoc sensitivity analyses (data not shown) revealed respondents which completed the survey via telephone implemented significantly more healthy eating (p < 0.01) and physical activity (p < 0.01) practices than those completing the survey via online. These findings suggest social desirability bias may have occurred to some extent, particularly by telephone respondents. Therefore, our findings potentially overestimate the actual implementation of practices assessed. Future research should seek to use items validated in this setting, include lie detection or control questions. Where there is adequate resourcing, this could be supplemented with observational or audit methods. Finally, data collection occurred during the context of COVID-19. As such, practice implementation may reflect constraints due to periods of lockdowns across Australia.

## 5 Conclusion

This study provides necessary estimates of the prevalence of implementation of evidence-based healthy eating and physical activity practices in centre-based ECEC services across Australia. Broadly, this study found the prevalence of implementation of healthy eating and physical activity practices in Australian ECEC services to be adequate, however, there continues to be specific evidence-based practices which are not being fully implemented by most services, particularly around educator training and provision of workshops to families for both healthy eating and physical activity. Such practices should be considered for prioritisation for government investment. To ensure population-level improvements in child healthy eating and physical activity can be achieved, including meeting broader child-level guideline recommendations, home environments should also be supported to promote child healthy eating and physical activity. The statistically significant association between implementation of healthy eating practices by service type suggests preschools may require additional implementation support. These results can guide researchers, policy makers and health care practitioners in the development of strategies to improve the health outcomes of children attending ECEC services.

## Funding source

This study was funded by the NHMRC Centre for Research Excellence grant (CIA: Wolfenden, APP1153479). The contents are the responsibility of the authors and do not reflect the views of the NHMRC. AG is supported by a Heart Foundation Postdoctoral Fellowship (102518). LW was supported by a NHMRC Leadership Grant (APP1197022). SLY is supported by a Heart Foundation Future Leader Fellowship (106654). Hunter New England and the University of Newcastle provided infrastructure and in-kind support.

## Declaration of Competing Interest

The authors declare that they have no known competing financial interests or personal relationships that could have appeared to influence the work reported in this paper.

## Data Availability

Data will be made available on request.

## References

[b0005] Ajja R., Beets M.W., Chandler J., Kaczynski A.T., Ward D.S. (2015). Physical activity and healthy eating environmental audit tools in youth care settings: A systematic review. Prev. Med..

[b0010] Ambrosini G.L. (2014). Childhood dietary patterns and later obesity: a review of the evidence. Proc. Nutr. Soc..

[b0015] American Academy of Pediatrics APHA, National Resource Center for Health and Safety in Child Care and Early Education,. Caring for Our Children: National Health and Safety Performance Standards Guidelines for Early Care and Education Programs. 4th edition ed. Itasca, Illinois: American Academy of Pediatrics; 2019.

[b0020] Australian Bureau of Statistics. Australian Statistical Geography Standard (ASGS) Edition 3 2021 [2021]. Available from: https://www.abs.gov.au/statistics/standards/australian-statistical-geography-standard-asgs-edition-3/jul2021-jun2026].

[b0025] Australian Bureau of Statistics. Impact of lockdowns on household consumption - insights from alternative data sources: Australian Bureau of Statistics; 2021.

[b0030] Australian Bureau of Statistics. Preschool Education. In: Statistics ABo, editor. 2022.

[b0035] Australian Bureau of Statistics. 2033.0.55.001 - Census of Population and Housing: Socio-Economic Indexes for Areas (SEIFA), Australia, 2016: Australian Bureau of Statistics; 2018 [Available from: https://www.abs.gov.au/ausstats/abs@.nsf/Lookup/by%20Subject/2033.0.55.001∼2016∼Main%20Features∼IRSAD%20Interactive%20Map∼16].

[b0040] Australian Children's Education and Care Quality Authority. Guide to the national quality framework Sydney, NSW: Australian Children's Education and Care Quality Authority; 2018 [Available from: https://www.acecqa.gov.au/sites/default/files/2022-05/Guide-to-the-NQF-220511-compressed.pdf].

[b0045] Australian Children’s Education, Care Quality, Authority., 2021. National registers.

[b0050] Australian Children's Education and Care Quality Authority. NQF snapshot Q2 2022: A quarterly report from the Australian Children’s Education and Care Quality Authority. 2022.

[b0055] Australian Institute of Health and Welfare. Nutrition across the life stage. Cat. no. PHE 227. Canberra: AIHW; 2018.

[b0060] Banfield E.C., Liu Y., Davis J.S., Chang S., Frazier-Wood A.C. (2016). Poor Adherence to US Dietary Guidelines for Children and Adolescents in the National Health and Nutrition Examination Survey Population. J. Acad. Nutr. Diet..

[b0065] Barnes C., Yoong S.L., Wolfenden L., Nathan N., Wedesweiler T., Kerr J., Pearson N., Grady A. (2021). The Association between Australian Childcare Centre Healthy Eating Practices and Children's Healthy Eating Behaviours: A Cross-Sectional Study within Lunchbox Centres. Nutrients.

[b0070] Byrd-Williams C.E., Dooley E.E., Thi C.A., Browning C., Hoelscher D.M. (2019). Physical activity, screen time, and outdoor learning environment practices and policy implementation: a cross sectional study of Texas child care centers. BMC Public Health.

[b0080] Coleman B., Dyment J.E. (2013). Factors that limit and enable preschool-aged children’s physical activity on child care centre playgrounds. J. Early Child. Res..

[b0085] Department of Health and Aged Care. Physical activity and exercise guidelines for all Australians: For infants, toddlers and preschoolers (birth to five years). In: Department of Health and Aged Care, editor. 2021.

[b0090] Ernst J. (2014). Early childhood educators’ use of natural outdoor settings as learning environments: an exploratory study of beliefs, practices, and barriers. Environ. Educ. Res..

[b0095] Fox M.K., Gearan E., Cannon J., Briefel R., Deming D.M., Eldridge A.L., Reidy K.C. (2016). Usual food intakes of 2- and 3-year old U.S. children are not consistent with dietary guidelines. *BMC*. Nutrition.

[b0100] Green A.M., Mihrshahi S., Innes-Hughes C., O'Hara B.J., McGill B., Rissel C. (2020). Implementation of an Early Childhood Healthy Eating and Physical Activity Program in New South Wales, Australia: Munch & Move. Front. Public Health.

[b0105] Hidayat K., Zhou H.J., Shi B.M. (2020). Influence of physical activity at a young age and lifetime physical activity on the risks of 3 obesity-related cancers: systematic review and meta-analysis of observational studies. Nutr. Rev..

[b0115] Jackson J.K., Jones J., Nguyen H., Davies I., Lum M., Grady A., Yoong S.L. (2021). Obesity Prevention within the Early Childhood Education and Care Setting: A Systematic Review of Dietary Behavior and Physical Activity Policies and Guidelines in High Income Countries. Int. J. Environ. Res. Public Health.

[b0120] Jones J., Wolfenden L., Grady A., Finch M., Bolsewicz K., Wedesweiler T., Yoong S.L. (2020). Implementation of continuous free play schedules in Australian childcare services: A cross-sectional study. Health Promot. J. Austr..

[b0125] Little H., Wyver S. (2008). Outdoor Play: Does Avoiding the Risks Reduce the Benefits?. Australas. J. Early Childhood.

[b0130] Liu S.T., Graffagino C.L., Leser K.A., Trombetta A.L., Pirie P.L. (2016). Obesity Prevention Practices and Policies in Child Care Settings Enrolled and Not Enrolled in the Child and Adult Care Food Program. Matern. Child Health J..

[b0135] Lum M., Wolfenden L., Jones J., Grady A., Christian H., Reilly K., Yoong S.L. (2022). Interventions to Improve Child Physical Activity in the Early Childhood Education and Care Setting: An Umbrella Review. Int. J. Environ. Res. Public Health.

[b0140] Martínez-Mesa J., González-Chica D.A., Duquia R.P., Bonamigo R.R., Bastos J.L. (2016). Sampling: how to select participants in my research study?. An. Bras. Dermatol..

[b0145] Matwiejczyk L., Mehta K., Scott J., Tonkin E., Coveney J. (2018). Characteristics of Effective Interventions Promoting Healthy Eating for Pre-Schoolers in Childcare Settings: An Umbrella Review. Nutrients.

[b0150] National Health and Medical Research Council (2013).

[b0155] Neelon S.E., Burgoine T., Hesketh K.R., Monsivais P. (2015). Nutrition practices of nurseries in England. Comparison with national guidelines. *Appetite*..

[b0160] OECD. PF3.2: Enrolment in Childcare and Pre-School 2021 [Available from: https://www.oecd.org/els/soc/PF3_2_Enrolment_childcare_preschool.pdf].

[b0165] Public Health England (2018).

[b0170] Public Health England. Government Dietary Recommendations: Government recommendations for energy and nutrients for males and females aged 1 – 18 years and 19+ years. In: Health Do, editor. London, England: Public Health England; 2016.

[b0180] Sisson S.B., Campbell J.E., May K.B., Brittain D.R., Monroe L.A., Guss S.H., Ladner J.L. (2012). Assessment of food, nutrition, and physical activity practices in Oklahoma child-care centers. J. Acad. Nutr. Diet..

[b0185] Stacey F.G., Finch M., Wolfenden L., Grady A., Jessop K., Wedesweiler T., Bartlem K., Jones J., Sutherland R., Vandevijvere S., Wu J.H.Y., Yoong S.L. (2017). Evidence of the Potential Effectiveness of Centre-Based Childcare Policies and Practices on Child Diet and Physical Activity: Consolidating Evidence from Systematic Reviews of Intervention Trials and Observational Studies. Curr Nutr Rep..

[b0190] Tapia-Serrano M.A., Sevil-Serrano J., Sánchez-Miguel P.A., López-Gil J.F., Tremblay M.S., García-Hermoso A. (2022). Prevalence of meeting 24-Hour Movement Guidelines from pre-school to adolescence: A systematic review and meta-analysis including 387,437 participants and 23 countries. J. Sport Health Sci..

[b0195] Tucker P. (2008). The physical activity levels of preschool-aged children: A systematic review. Early Child. Res. Q..

[b0200] U.S. Department of Agriculture and U.S. Dietary Guidelines for Americans, 2020-2025. In: Services DoHaH, editor. 9th Edition ed., 2020.

[b0205] U.S. Department of Health and Human Services. Physical activity guidelines for Americans. In: Services USDoHaH, editor. 2nd edition ed. Washington, DC 2018.

[b0210] UK Chief Medical Officer (CMO) Guidelines Writing Group. UK Chief Medical Officers' physical activity guidelines. 2019.

[b0215] Whitaker R.C., Gooze R.A., Hughes C.C., Finkelstein D.M. (2009). A national survey of obesity prevention practices in Head Start. Arch. Pediatr. Adolesc. Med..

[b0220] Wolfenden L., Finch M., Nathan N., Weaver N., Wiggers J., Yoong S.L., Jones J., Dodds P., Wyse R., Sutherland R., Gillham K. (2015). Factors associated with early childhood education and care service implementation of healthy eating and physical activity policies and practices in Australia: a cross-sectional study. Transl. Behav. Med..

[b0225] World Health Organisation. Population-based approaches to childhood obesity prevention Geneva, Switzerland: World Health Organization; 2012.

[b0230] Yoong S, Lum M, Jackson J, Wolfenden, Barnes C, Hall A, et al. Healthy eating interventions delivered in early childhood education and care settings for improving the diet of children aged six months to six year. *Cochrane Database of Sys Rev*. in press.

[b0235] Yoong S.L., Finch M., Nathan N., Wiggers J., Lecathelinais C., Jones J., Dodds P., Wolfenden L. (2016). A longitudinal study assessing childcare services' adoption of obesity prevention policies and practices. J. Paediatr. Child Health.

