# Supplementary material for: Implementation of healthy eating and physical activity practices in Australian early childhood education and care services: A cross-sectional study
Source: Prev Med Rep. 2023 Oct 4;36:102455. doi: 10.1016/j.pmedr.2023.102455 (PMC10571023; doi:10.1016/j.pmedr.2023.102455)
Supplement: Supplementary data 1 [file mmc1.docx]

Appendix A. Survey items to assess implementation of healthy eating and physical activity practices

| Services only received items applicable to their service, based on previous responses. For all items, services had the option to select “prefer not to say” or “unsure”. |
| --- |

**Healthy eating**

| **Does your service provide meals and/or snacks to children?** |
| --- |
| 1 Yes, all meals and snacks |
| 2 Yes, some meals including main meals (eg. lunch) |
| 3 Yes, heat and serve meals are provided by an external company |
| 4 Yes, snacks only |
| 5 No, families provide all meals and snacks |

*Lunchbox services only*

| **Does your service provide a resource to families with recommendations for the types of food and drinks brought in lunchboxes from home?**  **This could be a checklist, a resource, or a list of foods that are recommended for families to pack in, or leave out, of their child’s lunchbox. Examples of resources include the Australian Dietary Guidelines, Caring for Children 2014 ‘Lunchbox Checklist for Food Brought from Home for 2-5 year olds’ or local government or Health District produced resources.** |
| --- |
| 1 Yes, service provides this to all parents |
| 2 No, service does not provide any resources to parents |
| **Do these resources refer to the Australian Dietary Guidelines?** |
| 1 Yes |
| 2 No |
| **How often does your service observe children’s lunchboxes to ensure that they are consistent with the Australian Dietary Guidelines?** |
| 1 Every day |
| 2 3-4 times per week |
| 3 1-2 times per week |
| 4 Once per week or less |
| 5 Service observes, but without reference to the Australia Dietary Guidelines |
| 6 Service doesn’t observe |
| **If a lunchbox is not consistent with the Australian Dietary Guidelines, how often does the service provide feedback to families?**  **Examples of feedback may include: verbal or written , delivered via newsletters or other resources reminders to families of the service lunchbox guidelines; including common non-consistent foods items in the newsletter/family notice boards with ideas for healthier swaps; or, through parent/family information sessions** |
| 1 Never |
| 2. Daily |
| 3. At least weekly |
| 2 At least monthly |
| 3 Quarterly (each term) or less often |
| 4 Not applicable. Lunchbox is always consistent |

*Menu services only*

| **Is your service involved in planning the menu?**  **By having a role in planning the menu, this means that someone at the service (for example the service cook, service director or yourself) has a direct role in making decisions about the menu such as: deciding what items to include, changing the menu based on feedback, ordering food items, promoting the menu to families, monitoring the budget, etc.** |
| --- |
| 1 Yes |
| 2 No |
| **Has your service cook completed training in planning, preparing and serving nutritious meals and snacks?**  **Training may include the Munch & Move Healthy Menu Planning Workshop in NSW or TAFE or other training courses which include the unit, ‘Promote and provide healthy food and drinks’** |
| 1 Yes |
| 2 No |
| **Does the planner/service cook use any nutrition guidelines to plan their menus?**  **For example, Caring for Children Guidelines (NSW), Menu planning guidelines for long day care (Vic), Long Day Care Menu Planner (NT), Start them Right (Tas), Australian Dietary Guidelines** |
| 1 Yes (please specify) |
| 2 No |
| **Does the planner/service cook receive any of the following support from their service to plan their menus?** |
| 1 Support from external experts (e.g. health promotion team, dietitian) |
| 2 Network or professional networking opportunities |
| 3 Peer-learning or mentoring |
| 4 Written resources (e.g. recipes, books) |
| 5 Updated guidelines/nutrition recommendations |
| 6 Other (please specify) |
| **Do you have a separate menu for children aged 6 months to 2 years?** |
| 1 Yes |
| 2 No |
| **Please estimate the number of serves of fruit provided per child per day. Please provide your best guess where possible.**  **An example of one serve is one medium-sized banana or apple, or two small fruits such as apricots or plums or one cup of diced or canned fruit with no added sugar. Please think of the quantity served throughout the day. For example, if your services provides half an apple per child for morning tea and half an apple for afternoon tea, this equals to one serve.**  **Remember, these refers to children aged 6 months to 2 years** |
| 1 <1 |
| 2 1 |
| 3 2 |
| 4 3 |
| 5 4 |
| 6 5 or more |
| **Please estimate the number of serves of vegetables provided per child per day. Please provide your best guess where possible.**  **An example of one serve is a half cup of cooked green or orange vegetables, beans or peas, one cup of green leafy or raw salad vegetables or one medium tomato. Please think of the quantity served throughout the day. For example, if your services provides a half cup of raw salad vegetables per child for morning tea and a half cup of raw vegetables for afternoon tea, this equals to one serve.**  **Remember, these refers to children aged 6 months to 2 years.** |
| 1 < 1 |
| 2 1 |
| 3 2 |
| 4 3 |
| 5 4 |
| 6 5 or more |
| **The next questions refer to children aged 2-6 years.**  **Please estimate the number of serves of fruit provided per child per day. Please provide your best guess where possible.**  **An example of one serve is one medium-sized banana or apple, or two small fruits such as apricots or plums or one cup of diced or canned fruit with no added sugar Please think of the quantity served throughout the day. For example, if your services provides half an apple per child for morning tea and half an apple for afternoon tea, this equals to one serve. Remember, these refer to children aged 2-6 years** |
| 1 <1 |
| 2 1 |
| 3 2 |
| 4 3 |
| 5 4 |
| 6 5 or more |
| **How many times per day does your service provide vegetables on the menu?**  **Vegetables include fresh, frozen and tinned varieties, as well as legumes and beans BUT NOT INCLUDING chips, French fries or potatoes that are cooked in butter, cream or oil. An example of one serve is a half cup of cooked green or orange vegetables or dried or canned beans or one cup of green leafy or raw salad vegetables. Remember, these refers to children aged 2-6 years.** |
| <Record total number> |
| **Please estimate the number of serves of vegetables provided per child per day. Please provide your best guess where possible.**  **An example of one serve is a half cup of cooked green or orange vegetables, beans or peas, one cup of green leafy or raw salad vegetables or one medium tomato. Please think of the quantity served throughout the day. For example, if your services provides a half cup of raw salad vegetables per child for morning tea and a half cup of raw vegetables for afternoon tea, this equals to one serve. Remember, these refer to children aged 2-6 years.** |
| 1 <1 |
| 2 1 |
| 3 2 |
| 4 3 |
| 5 4 |
| 6 5 or more |
| **In the past two weeks did your service provide any of the following foods for snacks or at main meals?**  **Select all that apply. Remember, these refer to children aged 2-6 years** |
| 1 Confectionary, chocolate, ice cream |
| 2 Processed meats (e.g. sausages, devon, frankfurts, cabanossi, bacon, salami) |
| 3 Iced or creamed cakes, lamingtons, slices, sweet pastries or donuts |
| 4 Wholegrain or rice crackers or rice cakes |
| 5 Potato chips, corn chips, cheese flavoured snacks (e.g. Twisties) |
| 6 Flavoured biscuits/crackers (e.g. Shapes, Jatz, Country Cheese) |
| 7 Roll ups or fruit sticks |
| 8 Sweet biscuits (e.g. Tiny Teddies, gingerbread babies, Oreo wafer sticks) |
| 9 Jelly or flavoured custard or puddings (e.g. Yogo, chocolate snack pack) |
| 10 Muesli bars or breakfast bars (e.g. LCMs, Milo bars, Nutri-grain bars) |
| 11 Jam, cream cheese, vegemite, nutella |
| 12 None of the above |

*Both menu and lunchbox services*

| **What drink(s) does your service provide to children?**  **Select all that apply. This refers to children aged 2-6 years.** |
| --- |
| 1 Fruit juice or fruit drink including 100% fruit juice |
| 2 Cordial |
| 3 Water |
| 4 Full cream milk |
| 5 Reduced fat milk (including lite or low fat milk) |
| 6 Flavoured milk |
| 7 Soft drink |
| 8 Other (please specify) |
| 9 No drinks provided |
| **How often does your service implement strategies to encourage children to consume age appropriate beverages including water and milk?**  **This may include role modelling drinking water or milk, praising children for drinking water or milk, suggesting “water breaks”.** |
| 1 Daily (or every day the service is open) |
| 2 2-4 days per week |
| 3 Once per week |
| 4 Less than once per week |
| **Does your service undertake any planned healthy eating education lessons (not including meal time)?** |
| 1 Yes, daily |
| 2 Yes, weekly |
| 3 Yes, monthly |
| 4 Yes, less than monthly |
| 5 No, not at all |
| **Does your service undertake any interactive, experiential healthy eating activities (eg cooking lessons and discussions around food growing etc)?** |
| 1 Yes, daily |
| 2 Yes, weekly |
| 3 Yes, monthly |
| 4 Yes, less than monthly |
| 5 No, not at all |
| **Does your service intentionally expose children to different vegetables as part of experiential learning?** |
| 1 Yes, daily |
| 2 Yes, weekly |
| 3 Yes, monthly |
| 4 Yes, less than monthly |
| 5 No, not at all |
| **Does your service undertake any play-based healthy eating activities (eg. use of puppets, mascot or other resources to communicate messages around healthy eating)?** |
| 1 Yes, daily |
| 2 Yes, weekly |
| 3 Yes, monthly |
| 4 Yes, less than monthly |
| 5 No, not at all |
| **How often do staff model, reinforce and implement healthy eating and nutrition practices with children during mealtimes?**  **For example, sitting with the children during meal times, eating healthy foods in front of the children, talking with the children about the foods they are eating, encouraging children to try foods that are new to them.** |
| 1 Daily (or every day the day the service is open) |
| 2 At least weekly |
| 3 At least monthly |
| 4 Less than monthly |
| 5 Never |
| **In the past year, did you offer healthy eating education to families in the form of parent/families workshops or meetings?**  **These may be offered in person or online.** |
| 1 Yes, to all parent/families |
| 2 Yes, to parent/families of 3-6 year olds only |
| 3 Yes, to parent/families of 0-2 year olds only |
| 4 No |
| **In a typical year, how many of your primary contact educators participate in professional development or training which promotes healthy eating for children? This may include face to face or online training.**  **Primary contact educators include both full-time and part-time educators.** |
| 1 All |
| 2 Almost all |
| 3 About half |
| 4 Less than half |
| 5 None |
| **In the past year, did the service provide educators with any of the following additional support or resources to increase healthy eating for children?**  **Examples include onsite visits, group meetings, refresher training, newsletters or other written resources.** |
| 1 Peer-support meetings with colleagues |
| 2 Refresher training |
| 3 Newsletters |
| 4 Feedback from your service or other organisations |
| 5 Telephone support |
| 6 Other (please specify) |
| 7 None of the above |
| **Does your service have a written nutrition policy?** |
| 1 Yes |
| 2 No |
| **Does your nutrition policy include any of the following elements:**  **Select all that apply.** |
| 1 Strategies are in place to ensure that foods provided by families is consistent with the Australian Dietary Guidelines (lunchbox services only) |
| 2 Food provided by your service is consistent with the Australian Dietary Guidelines (menu services only) |
| 3 Sugar-sweetened beverages are not available to children, or there is a separate sugar sweetened beverage policy |
| 4 Strategies are in place to ensure food isn’t used as a reward or incentive for children |
| 5 Educators role model healthy food and drink choices |
| 7 Creating healthy mealtime environments |
| 8 Educator practices to encourage healthy eating |
| 9 Planned and informal nutrition education for children |
| 10 Professional development on child nutrition |
| 11 Guidelines for foods offered during holidays and celebrations |
| 12 None of the above |
| **Does your beverage/drinks policy include any of the following elements:**  **Select all that apply.** |
| 1 Supporting water as a drink for children aged 3-5 years |
| 2 Drinks provided/allowed by your service are consistent with the Australian Dietary Guidelines or Caring for Children Guidelines and age appropriate |
| 3 Sugar-sweetened beverages are not available to children |
| 4 Educators role model healthy drink choices |
| 5 None of the above |

**Physical activity**

| **Thinking back to the last week, for the room/s with the highest number of 3-6 year olds, how much time in total is provided for unstructured child-initiated free-play per day?**  **By free play we mean whenever structured (intentional) or educator-led activities are not in place, and children are able to choose what type of activity they do and explore the surrounding environment as they like. This includes indoor-only, outdoor-only and indoor-outdoor free play. Please give your best estimate or an overall average if this varies day to day.** |
| --- |
| <Total number minutes> |
| **Of the total time provided for child-initiated free play, how much of that time was for indoor-only free play per day?**  **Child-initiated free play refers to the opportunity for children to engage in unstructured, voluntary and child-initiated activities, without adults leading them. Remember, these are for the room with the most 3-6 year olds.** |
| <Total number minutes> |
| **Of the total time provided for child-initiated free play, how much of that time was for outdoor-only free play per day?** |
| <Record total number minutes> |
| **Of the total time provided for child-initiated free play, how much of that time was for indoor-outdoor free play per day?**  **By indoor-outdoor free play we mean children are allowed to access and move freely between indoor and outdoor environments, whenever a structured (intentional) or educator led activity is not in place. Some services refer to this as “free-flow” free play.** |
| <Record total number minutes> |
| **Thinking back to the last week, how much time in total did children spend participating in structured (intentional) or educator-led physical activities per day?**  **Educator led activities include circle time, music, dancing or planned activities to develop complex motor skills and movement patterns.** |
| <Total number minutes> |
| **Thinking about the last week, how many days a week did your service provide an activity designed to intentionally teach and develop the various fundamental movement skills for children aged 3-6 years of age? This could be during a transition activity, group or circle time, or during outdoor play.**  **Fundamental movement skills and movement patterns refer to basic gross motor movement skills such as running, catching, jumping and kicking.** |
| 1 Never |
| 2 One day |
| 3 Two days |
| 4 Three days |
| 5 Four days |
| 6 Daily (or every day the service is open) |
| **How often does your service have portable play equipment available and in good condition for children to use?**  **For example, balls, rope, hula hoops, floor mats and parachutes.** |
| 1 Always |
| 2 Often |
| 3 Sometimes |
| 4 Rarely |
| 5 Never |
| **In your opinion, is there sufficient portable play equipment for children to share?** |
| 1 Yes |
| 2 No (please specify) |

| **How often does your service re-organise the outdoor play area?** |
| --- |
| 1 Almost never |
| 2 Once a month |
| 3 Once every two weeks |
| 4 At least once a week |
| 5 Multiple times per week |
| 6 Every day (or every day the service is open) |
| **How often do educators in your service model active play when children are outside?** |
| 1 Almost never |
| 2 Once a month |
| 3 Once every two weeks |
| 4 At least once a week |
| 5 Multiple times per week |
| 6 Every day (or every day the service is open) |
| **How often does your service plan specific activities for outdoor time?** |
| 1 Almost never |
| 2 Once a month |
| 3 Once every two weeks |
| 4 At least once a week |
| 5 Multiple times per week |
| 6 Every day (or every day the service is open) |
| **How often do educators in your service engage children in active play?** |
| 1 Almost never |
| 2 Once a month |
| 3 Once every two weeks |
| 4 At least once a week |
| 5 Multiple times per week |
| 6 Every day (or every day the service is open) |
| **In the past year, did you offer physical activity education to families in the form of parent/families workshops or meetings? These may be offered in person or online.** |
| 1 Yes, to all parent/families |
| 2 Yes, to parent/families of 3-6 year olds only |
| 3 Yes, to parent/families of 0-2 year olds only |
| 4 No |
| **In the past year, how many of your primary contact educators participated in training which promotes physical activity for children? This can include online or face to face training**  **Primary contact educators include both full-time and part-time educators** |
| 1 All |
| 2 Almost all |
| 3 About half |
| 4 Less than half |
| 4 None |
| **In the past year, did your service provide educators with any of the following additional support or resources to increase physical activity for children?**  **Select all that apply** |
| 1 Peer-support meetings with colleagues |
| 2 Refresher training |
| 3 Newsletters |
| 4 Feedback from your service or other organisations |
| 5 Telephone support |
| 6 Other (please specify) |
| **Does your service have a written policy, procedure or guideline encouraging physical activity?** |
| 1 Yes, independent policy, guideline or procedure |
| 2 Yes, integrated within another policy, guideline or procedure. |
| 3 No |
| **Does your physical activity policy, procedure or guideline include the following elements:** |
| 1 Reference to the Australian 24-Hour Movement Guidelines for the Early Years (Birth to 5 years) |
| 2 Physical activity is embedded in the daily curriculum through spontaneous and intentionally planned active play time that is both child initiated and educator led |
| 3 Educators actively role model to children appropriate physical activity behaviours |
| 4 Professional development on children’s physical activity |
| 5 Education for families on children’s physical activity |
| 6 None of the above |
